# Metabolic Phenotyping of Anks3 Depletion in mIMCD-3 cells - a Putative Nephronophthisis Candidate

**DOI:** 10.1038/s41598-018-27389-y

**Published:** 2018-06-13

**Authors:** Manuel Schlimpert, Simon Lagies, Vadym Budnyk, Barbara Müller, Gerd Walz, Bernd Kammerer

**Affiliations:** 1 Center for Biological Systems Analysis, Albert-Ludwigs-University of Freiburg, Freiburg, Germany; 2 Spemann Graduate School of Biology and Medicine, Albert-Ludwigs-University of Freiburg, Freiburg, Germany; 3 Faculty of Biology, Albert-Ludwigs-University of Freiburg, Freiburg, Germany; 4 Department of Medicine, Renal Division, Albert-Ludwigs-University of Freiburg, Medical Center, Freiburg, Germany; 5 University of Freiburg, BIOSS Center for Biological Signaling Studies, Freiburg, Germany

## Abstract

Nephronophthisis (NPH) is an autosomal recessive form of cystic kidney disease and the leading cause of hereditary kidney failure in children and young adults. Like other NPH proteins, the NPHP16/Anks6-interacting protein Anks3 has been identified to cause laterality defects in humans. However, the cellular functions of Anks3 remain enigmatic. We investigated the metabolic impact of Anks3 depletion in cultured murine inner medullary collecting duct cells via GC-MS profiling and LC-MS/MS analysis. Combined metabolomics successfully identified 155 metabolites; 48 metabolites were identified to be significantly altered by decreasing Anks3 levels. Especially, amino acid and purine/pyrimidine metabolism were affected by loss of Anks3. Branched-chain amino acids were identified to be significantly downregulated suggesting disrupted nutrient signalling. Tryptophan and 1-ribosyl-imidazolenicotinamide accumulated whereas NAD^+^ and NADP^+^ concentrations were diminished indicating disturbances within the tryptophan-niacin pathway. Most strikingly, nucleosides were reduced upon Anks3 depletion, while 5-methyluridine and 6-methyladenosine accumulated over time. Hence, elevated PARP1 and cleaved PARP1 levels could be detected. Furthermore, living cell number and viability was significantly declined. In combination, these results suggest that Anks3 may be involved in DNA damage responses by balancing the intracellular nucleoside pool.

## Introduction

Nephronophthisis (NPH) is a congenital form of cystic kidney disease that causes kidney failure in childhood and adolescence^1–3^. With an incidence of 1 in 50,000 to 1 in 900,000 births, it is the most common genetic cause of end stage renal disease (ESRD) in the first decades of life^4^. In contrast to classical polycystic kidney diseases, NPH is characterised by normal or slightly reduced kidney size and tubulointerstitial fibrosis^1^. In addition, cysts are formed within the corticomedullary junction^1^. Early symptoms of NPH are usually diagnosed in children of six years of age developing polyuria, polydipsia and anaemia, which result from a urinary concentration defect^2^. However, effective treatment preventing progression into cyst formation and ESRD is currently lacking^5^.

Currently, mutations in more than 20 different genes are known to individually cause NPH and closely related diseases. However, these mutations only explain around 40% of clinical manifestations^6,7^. The corresponding proteins are mostly localised to the primary cilium, a crucial sensing and signalling organelle protruding into extracellular environment^8^. How structural or functional defects in cilia cause NPH and related ciliopathies has been subject to intense study. Disturbed signalling pathways, in particular mTOR signalling, have been emerged as key factors in the progression of polycystic kidney disease^9,10^. However, the chain of molecular events triggering NPH development remains unclear^11^. Interestingly, recent findings indicate that several pathways of central cellular metabolism are deregulated in kidneys of animal models of autosomal-dominant polycystic kidney disease^12,13^. This raises the intriguing possibility that perturbation of metabolic networks may also be involved in the molecular pathogenesis of NPH.

Recent findings revealed that a defectively mutated Anks3 is causing laterality defects in an autosomal recessive manner^14^. Furthermore, recent studies revealed Anks6 as a strong interaction partner of Anks3^15^. Interestingly, Anks6 mutations in humans cause NPH with similar phenotypes in animal models^16–18^. In addition, Anks3 was shown to be phosphorylated dependent on NEK7 hence preventing nuclear localisation of NEK7^19^. It has been shown that both Anks3 as well as Anks6 are interacting with Bicaudal-C homologue 1 (Bicc1) which is mainly expressed in liver, pancreas and kidney functioning as negative regulator for canonical Wnt signalling^20–23^. Furthermore, interaction of Anks3 and Anks6 with hypoxia-induced factor 1 alpha inhibitor (HIF1AN) was previously described^16,23^. HIF1AN, also called factor inhibiting HIF1α (FIH), serves as oxygen sensor by inhibiting signalling of VHL/HIF1α under normoxic conditions^24,25^. Recent findings suggest that Anks3 is a potential substrate for hydroxylation by HIF1AN^23^. Given that Anks3 is not only required for ciliary motility and polarity^23^ but also interacting with important intracellular signalling cascades such as WNT signalling, we hypothesise that defects of Anks3 might influence central cellular metabolism dependent on intact mitochondrial function for energy and biomass production.

The aim of the present study was to investigate to which extend loss of Anks3 affects central cellular metabolism such as glycolysis, TCA cycle and amino acid metabolism. Therefore usage of gas-chromatography coupled to mass spectrometry (GC-MS) provides an excellent opportunity to monitor central metabolism in a global untargeted manner. Thus, murine inner medullary collecting duct (mIMCD3) cells were analysed by GC-MS after induction of Anks3 depletion to address alterations occurring by direct disturbance of NPH protein candidates. Targeted liquid chromatography tandem mass spectrometry (LC-MS/MS) analyses were conducted to specifically monitor free amino acids, purine/pyrimidine metabolites as well as nucleosides.

## Materials and Methods

### Cell Lines

**Figure 1:**
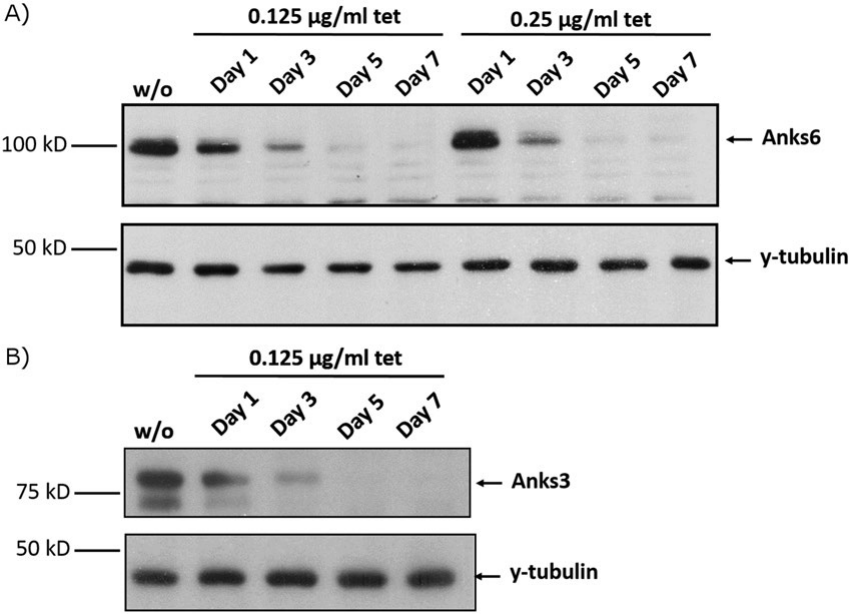
Western Blot analysis of Anks6 and Anks3 protein levels. Boxed areas indicate individual western blot exposures. Protein of interest and loading control are stained on the same blot. (**A**) Successful knockdown induction of Anks6 at tetracycline concentrations below 0.5 µg/mL. Diminished signal after 5 days of tet treatment. (**B**) Validation of Anks3 knockdown, tet was used at a concentration of 0.125 µg/mL, knockdown was robustly detected after 5 days of tet treatment.

Murine inner medullary collecting duct (mIMCD3) cells were used to establish a tetracycline-inducible Anks3 knockdown cell line. The cells were lentivirally transduced with shRNA targeting the coding sequence for mAnks3. The knockdown was verified by Western blot analysis (Fig. 1B). The corresponding mIMCD3 control cell line tTR KRAB Luci-i was obtained by transducing mIMCD3 tTR KRAB cells with shRNA specific for luciferase. Sequences were tested for off target effects in silico using SciDirect software and web-based nucleotide blast tool.

### Cell Culture

All cell culture experiments were performed in five replicates. Four replicates were used for mass spectrometric metabolome analysis and one was used to verify the knockdown efficiency by western blot. For each condition 0.5 × 10^6^ cells were seeded onto 100 mm × 20 mm cell culture dishes (Corning, Kaiserslautern, Germany), containing 10 mL DMEM/F12 (Lonza, Cologne, Germany) media supplemented with 10% foetal bovine serum (Day −1). Cells were allowed to attach overnight before treatment. Media were changed regularly starting treatment with 0.125 µg/mL tetracycline (Sigma-Aldrich, Munich, Germany) at day 0, 2, 4 and 6, respectively (Supplementary Table S1).

At day 7, cells were washed twice with 0.9% sodium chloride (Sigma-Aldrich, Munich, Germany). Metabolism was quenched by addition of 1.5 mL ice-cold extraction buffer (90% methanol in water) containing 1 µg/mL β-phenylglucose, ribitol and 5 µg/mL isoguanosine as internal standards (Sigma-Aldrich, Munich, Germany) for 1 min on ice. Cells were lysed by extensive scraping with Corning® cell scrapers (Sigma-Aldrich, Munich, Germany). Cell extracts were collected in screw-cap tubes (Sarstedt AG, Nürnbrecht, Germany) containing 300 mg glass beads (Sigma-Aldrich, Munich, Germany) and stored in liquid nitrogen immediately^26^.

### Seahorse Analysis

For analysis of oxygen consumption rate (OCR) and extracellular acidification rate (ECAR), cells were treated for six days before analysis to establish the Anks3 knockdown as described above. 3 × 10^5^ cells/well were seeded onto a Seahorse XF miniplate. Basal respiration was measured at the beginning of the analysis. Subsequent inhibition of ATP synthase by oligomycin (1 µM, Sigma-Aldrich, Munich, Germany) enables measurement of non-mitochondrial respiration plus physiological proton leakage. After uncoupling of oxygen consumption and ATP production by treatment with 1 µM carbonyl-cyanide-4-(trifluoromethoxy)phenylhydrazone (FCCP, Sigma-Aldrich, Munich, Germany), maximum respiration capacity was acquired. Inhibition of respiratory chain complex I and III with 0.5 µM rotenone (Abcam, Cambridge, United Kingdom) and 0.5 µM antimycin A (Sigma-Aldrich, Munich, Germany) enabled the acquisition of non-mitochondrial respiration. Measured values were normalised to 1000 cells.

### Cell Growth and Viability

5 × 10^5^ cells were seeded after 7 days of knockdown induction. For determination of cell number and living cells, adherent cells were detached after 3 and 4 days of further tet treatment by addition of 1 mL 0.25% Trypsin/EDTA (Sigma-Aldrich, Munich, Germany) for 5 min at 37 °C. Trypsin reaction was stopped by 4 mL cell culture medium. 10 µL cell suspension was mixed with 10 µL trypan blue (Sigma-Aldrich, Munich, Germany). For cell counting with an automated cell counter (Biorad, Munich, Germany), ready-to-use counting chambers (Biorad, Munich, Germany) were loaded with 10 µL of staining cell suspension. Number of total cells was determined. Dead cells were trypan blue positive. Living cells were calculated by subtraction of total cell number with trypan blue positive cell number.

### Ki-67 staining

Cells were permeabilised at room temperature for 45 min. Cells were stained with Pe-Cy7 anti-mouse Ki-67 antibody for 45 min at 4 °C. Living cells were selected by forward and sideward scatter gating. A IgG1 κ isotope control was used to discriminate between ki-67 positive and negative cells (Supplementary Figure 8).

### Western Blot Analysis

To verify the knockdown, cells were lysed in lysis buffer containing 1% Triton-X100, 20 mM TRIS pH 7.5, 50 mM NaCl, 50 mM NaF, 15 mM Na_4_P_2_O_7_ and 0.1 mM EDTA, supplemented with Complete Protease Inhibitor Cocktail (Roche) and 1 mM sodium orthovanadate.

Lysates were cleared (16000 × *g*, 30 min, 4 °C), and after protein measurement (DC Protein Assay BIORAD) analysed by western blot (45 µg total protein/lane). As antibodies, anti-Anks3 pAb rabbit (Sigma), anti-PARP1 and monoclonal anti-γ-tubulin clone GTU88 (Sigma) were used.

### Metabolite Extraction

Polar metabolite extracts were obtained by homogenising cell extracts using a Precellys tissue homogeniser (Bertin Technologies, Montigny le Bretonneux, France). Cells were disrupted by three 15 s operating cycles at 6500 rpm, interrupted by 10 s breaks. Operating temperature was −10 °C. Cell debris and protein precipitates were removed by centrifugation (20000 × *g*, 4 °C and 5 min). The metabolite-containing supernatants were dried after transfer to new reaction tubes using a Concentrator plus vacuum rotator (Eppendorf AG, Hamburg, Germany) and stored under nitrogen atmosphere at −80 °C.

### GC-MS Analysis

For GC-MS analysis, metabolites were derivatised by reaction with 20 µL methoxyamine in pyridine (20 mg/mL) followed by 50 µL MSTFA (Sigma-Aldrich, Munich Germany) as previously described^26,27^ and transferred to crimp cap vials (VWR International, Darmstadt, Germany). Chemical blanks were prepared by the same procedure, using empty tubes. Previously derivatised samples were splitlessly injected (1 µL) into an Agilent 7890 A/5975 C system (Agilent Technologies, Waldbronn, Germany) equipped with an MPS 2 XL autosampler (Gerstel, Mülheim an der Ruhr, Germany). Chromatographic separation was carried out on a 60 m × 0.25 mm × 0.25 µm HP-5MS capillary column (Agilent, Waldbronn, Germany), using helium as carrier gas at a flow rate of 1 mL/min and a temperature program as follows: 80 °C for 3 min, 5 °C/min to 325 °C, 325 °C held for 14 min. Total runtime was 66 min, during which full-scan mass spectra (m/z 50–800) were acquired at a scan rate of 1.99s^−1^. Equilibration time and post run time were set to 1 min. Inlet temperature and temperature of MS source was set to 230 °C. Temperature of the quadrupole analyser was set to 150 °C at high vacuum (6.31 × 10^−6^ Pa). Septum purge flow was set to 3 mL/min. Perfluorotributylamine was used for previous mass calibration. For calculation of Kovat’s retention indices, a C10–C40 *n*-alkane standard mixture (Neochema, Bodenheim, Germany) was used as previously described^26^.

### GC-MS Data Analysis

For GC-MS analysis, peak identification and deconvolution was performed by application of *A*utomated *M*ass spectral *D*econvolution and *I*dentification *S*ystem (AMDIS, version 2.72) and NIST MS SEARCH (Version 2.2)^28^ with following parameters: component width 12, omit TIC, adjacent peak subtraction One, resolution medium, sensitivity medium, shape requirements medium. Processed samples were saved as ELU files allowing further processing with the online service SpectConnect^29^. Following SpectConnect settings were used for peak alignment: elution threshold 1.0 min, support threshold medium (component found in over 50% of replicates), similarity threshold of mass spectra over 80%.

Features were compared to following mass spectral databases: FiehnLib^30^, golmDB^31^ and NIST Mass Spectral Library. Match factor threshold was set to 750 with retention index deviation of <5% for sufficient compound annotation. Peak intensity areas of multiple annotated metabolites are summarised to yield the absolute peak intensity of each metabolite.

Peak intensity areas were normalised by division to peak intensity areas of β-phenylglucose as internal standard. Additionally, normalisation was performed by division of total peak areas of the chromatogram as an approximation for total cell number^32^ and subtraction of blank values^26^. Finally each variable was mean-centred and divided by the range of each variable called range scaling^33–38^.

### LC-MS/MS Analysis

For LC-MS/MS analysis, metabolite pellets were resuspended in 100 µL water. Samples were centrifuged for 5 min at 20000 × g and 4 °C. A pool samples serving as quality control were generated by combining 10 µL of each supernatant. 50 µL of each supernatant were transferred to LC-MS glass vials containing inserts for small volume injection (Agilent Technologies, Waldbronn, Germany). Injection volume was set to 10 µL. UPLC analysis was performed using an Agilent 1290 UPLC system (Agilent Technologies, Waldbronn, Germany). For separation of free amino acids as well as purines and pyrimidines a reverse-phase chromatography was applied on an Acquity UPLC HSS-T3 C18 (1.8 µm, 2.1 × 100 mm) column (Waters GmbH, Eschborn, Germany) using a solvent gradient of water +0.1% formic acid (Buffer A) and methanol +0.1% formic acid (Buffer B).

For separation of free amino acids, a flow rate of 0.2 mL/min was used and the column temperature was set to 50 °C. The following gradient was applied: 0–1.5 min, 0% B; 1.5–4 min, 0% to 5% B; 4–10 min, 5% to 95% B; 10–18 min, 95% B; 18–18.2 min, 95% to 0% B; 18.2–26 min, 0% B. Separation of purine and pyrimidine metabolites was achieved at a flow rate of 0.3 mL/min and a column temperature of 50 °C using following solvent gradient: 0–5 min, 0% B; 5–10 min, 0% to 30% B; 10–12 min, 30% to 98% B; 12–20 min, 98% B; 20–20.1 min, 98% to 0% B; 20.1–27 min, 0% B.

Mass spectra acquisition was performed on a 6460 triple quadrupole mass spectrometer (Agilent Technologies, Waldbronn, Germany) containing an electrospray ionisation source (ESI Jetstream, Agilent Technologies, Waldbronn, Germany). For amino acid analysis following MS settings were applied: capillary voltage: 3000 V (positive ionisation mode) or 3500 V (negative ionisation mode), nozzle voltage: 500 V (positive ionisation mode) or 300 V (negative ionisation mode), gas temperature: 350 °C (flow rate: 8 L/min), sheath gas temperature: 250 °C (flow rate: 5 L/min), nebulizer pressure: 30 psi. Purines and pyrimidines were analysed with following MS setting: capillary voltage: 4000 V (positive ionisation mode), nozzle voltage: 500 V (positive ionisation mode), gas temperature: 300 °C (flow rate: 7 L/min), sheath gas temperature: 350 °C (flow rate: 7 L/min), nebulizer pressure: 50 psi.

For semi-quantitative analysis dynamic multiple reaction monitoring (DMRM) scans were executed using a time filtering peak width of 0.07 min and cell accelerator voltages of 3 V (amino acid analysis) and 7 V (purine and pyrimidine analysis). Collision energies of standard substances were optimised using Mass Hunter Optimizer Software (Agilent Technologies, Waldbronn, Germany) (Supplementary Table S2).

### LC-MS/MS Data Analysis

Agilent MassHunter Qualitative Analysis B.08.00 and Agilent MassHunter Quantitative Analysis B.08.00 were used for processing of obtained raw data. For semi-quantitative analysis a minimal signal to noise ratio of 1:5 and a retention time deviation of 0.4 min were set as cut-off for peak integration. Quantifier MRM transitions were used for peak integration. Peak areas were divided by the internal standard isoguanosine. Blanks were subtracted and peak areas were further normalised by division of total peak areas of the chromatogram. Finally each variable was mean-centred and divided by the range of each variable, called range scaling^33–38^.

### Statistics

MetaboAnalyst was used as browser-based tool to perform statistical analysis based on R packages^33–38^. Hierarchical cluster analysis was performed with Pearson’s distance measurement and Ward’s minimum variance. One-way analysis of variance (ANOVA) was performed using a p-value cut-off 0.05 including multiple testing correction by false discovery rate (FDR) (Tukey test HSD) of 5% (q-value < 0.05). Principal component analysis (PCA) was used as unsupervised multivariate analysis to visualise global alterations between datasets. For comparison of D0 to D7 of conditions, student’s *t*-test was used with a corrected p-value (q-value) cut-off of 0.05 applying FDR correction.

The pathway figures were drawn in VANTED version 2.6.3^39^.

### Data Availability Statement

The datasets of this study is shown in Supplementary Table S3.

## Results and Discussion

### Optimisation of Anks3 Knockdown by Reduction of Tetracycline Concentrations

Identifying metabolic alterations dependent on disturbed protein expression requires a robust biological and analytical setup. It has been shown that tetracycline antibiotics can influence mitochondrial metabolism at higher concentrations than 0.5 µg/mL^40,41^. Therefore, tetracycline was titrated to lower concentrations to avoid side effects. Using the current protocol (Supplementary Table S1) tetracycline could be easily used at concentrations below 0.5 µg/mL as validated by western blot analysis (Fig. 1A). All following results were obtained by depletion of Anks3 using tetracycline at a concentration of 0.125 µg/mL (Fig. 1B).

### Statistical Analysis of Untargeted Metabolic Profiling

Untargeted metabolomics profiling yielded approximatly 800 unique mass spectral features. Application of defined filters and thresholds for feature annotation as described within the data analysis section provided a successful annotation of 92 metabolites (Supplementary Table S3).

**Figure 2:**
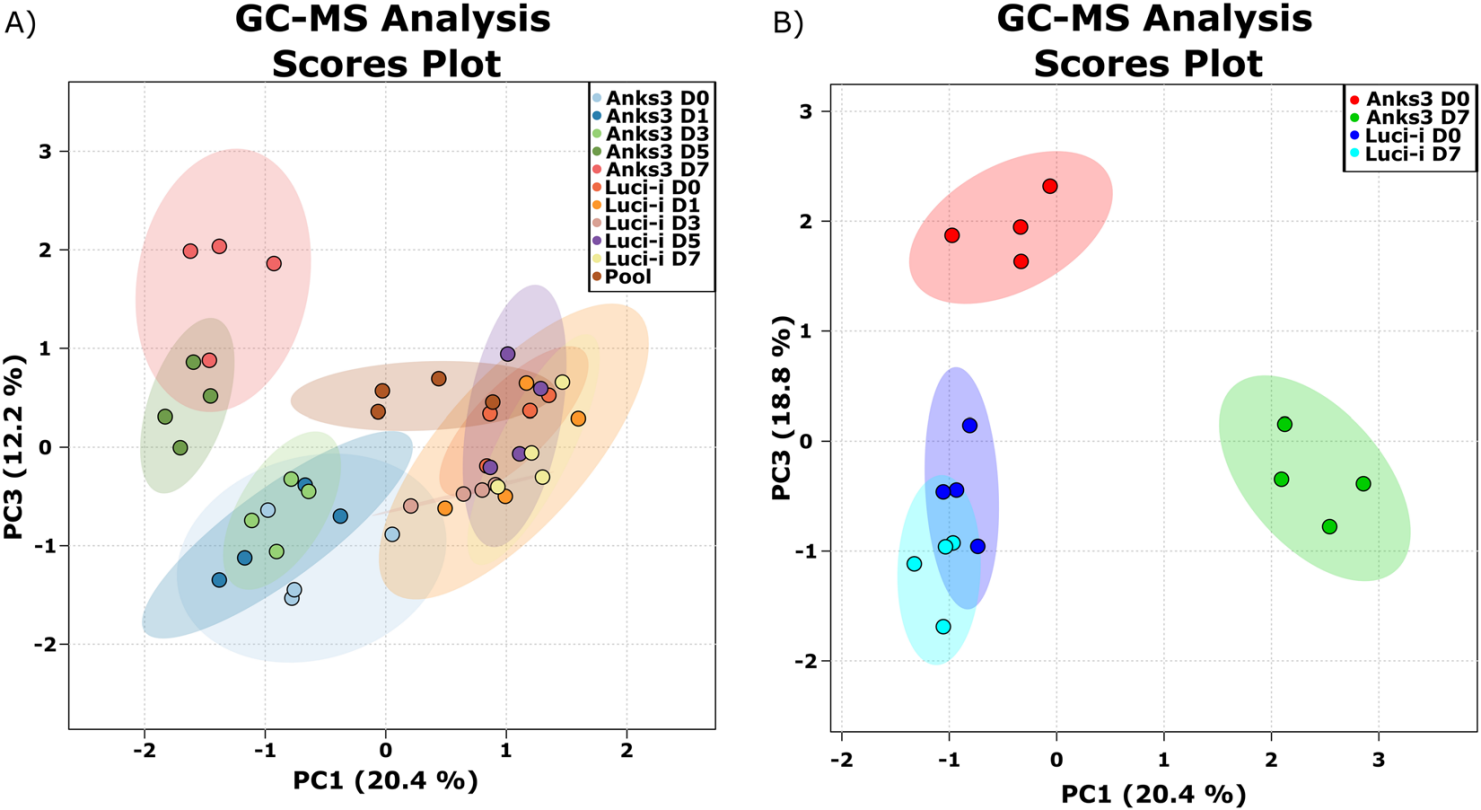
Principal Component Analysis of mIMCD3 krab shANKS3 and mIMCD3 krab shLuci-i conditions. (**A**) PCA containing all measured sample groups. Quality control samples are coloured in brown and labelled as Pool representing technical replicates of a mixture of all samples. Shaded areas highlight the 95% confidence interval of each condition. Separation was observed in mIMCD3 krab shANKS3 cells after five days of tet treatment. No separation occurred within the Luci-i control cell line. (**B**) PCA of D0 and D7 conditions. Shaded areas highlight the 95% confidence interval of each condition. Clear separation was observed between mIMCD3 krab shANKS3 cells after 7 days of tet treatment. Overlapping 95% confidence intervals were observed within the Luci-i control cell line even after 7 days of tet treatment.

**Figure 3:**
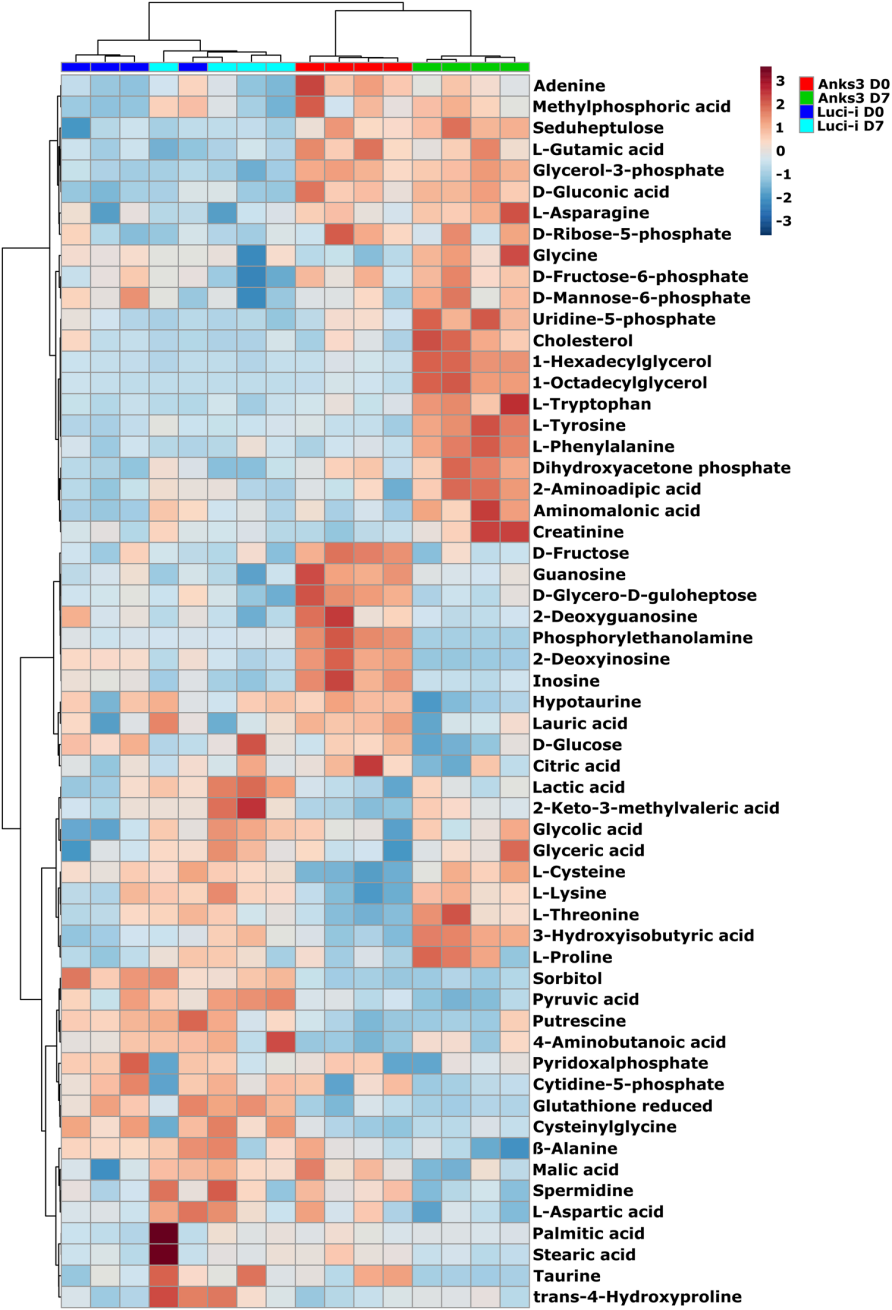
Heat map analysis of D0 and D7 conditions. Hierarchical clustering against Pearson and Ward yielded a separation of mIMCD3 krab shANKS3 conditions (D0 in red, D7 in green). Overlapped clustering of Luci-i controls highlights close similarity of both sample conditions and indicate less side effects of tet treatment on mIMCD3 cells in general. Higher abundant metabolites are depicted in red colours, lower abundant metabolites in blue. For heat map analysis, normalised peak intensities were range scaled to obtain a representative z-score suitable for heat map analysis, cluster analysis and PCA.

Annotated metabolite matrices were used for statistical analysis. One-way ANOVA was used in order to highlight significant changes within a multiple grouped experiment. Multiple testing correction revealed 58 components within the Anks3 experiment, respectively (Supplementary Table S4). However, ANOVA significance does not always reflect biological relevance. Therefore unsupervised principal component analysis was used to clarify whether there are global alterations leading so separation of different conditions (Fig. 2A). Treatment with tetracycline results only in slight alterations within treated and untreated Luci-i control conditions. However, significant separation occurred after 5 days of knockdown induction in the Anks3 knockdown conditions (Fig. 2A). Over 30% of total variation could be explained by using two principal components (Fig. 2A). As PCA and cluster analysis are not suitable to monitor whether certain metabolite classes are up- or downregulated, individual heat maps were generated to illustrate alterations of relative metabolite concentrations dependent on Anks3 knockdown (Fig. 3).

There are also differences occurring between untreated mIMCD3 shANKS3 cells compared to Luci-i control conditions which might be caused by viral transfection. However, focusing on alterations occurring exclusively upon knockdown induction revealed all biologically relevant metabolite changes caused by protein depletion (Figs 2 and 3).

### Metabolic Alterations Associated With Anks3 Depletion

As described in the previous section, focussing on metabolites which correlate with amount of detectable Anks3 protein yielded 27 metabolites to be significantly regulated comparing D0 to D7 conditions. In total, 14 metabolites were found to be upregulated, whereas 13 compounds were downregulated (Supplementary Table S5).

**Figure 4:**
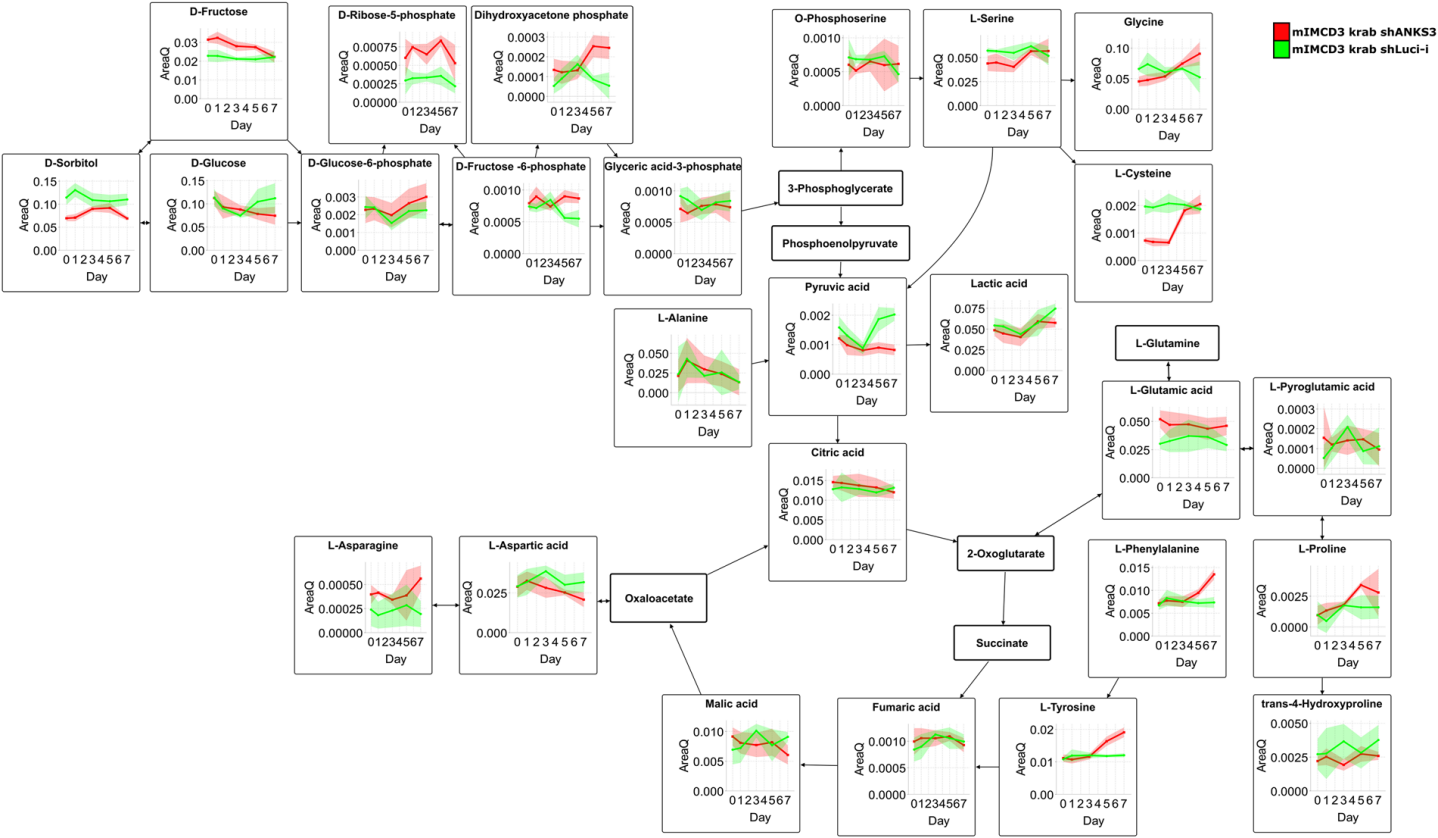
Pathway analysis of glycolysis and TCA cycle. Analysis of energy metabolites revealed no global alterations upon Anks3 protein depletion. However, within the energy metabolism fructose as well as pyruvic acid levels were found to be reduced. Amino acids were detected to be strikingly upregulated upon protein depletion. Especially, phenylalanine and tyrosine, but also cysteine and glycine were detected to be upregulated. mIMCD3 krab shANKS3 cells were represented in red. Luci-i controls were depicted in green. Shaded areas indicate the standard deviation at each time point.

As mentioned above, intermediates of energy and amino acid metabolism are robustly detectable by untargeted GC-MS profiling to yield an overview whether there are global alterations occurring. However, focussing on significantly regulated metabolites revealed reduced levels of pyruvate and fructose whereas all other component of glycolysis and TCA cycle were not significantly affected (Fig. 4). Further, analysis of oxygen consumption and extracellular acidification revealed no significant differences according to Anks3 depletion (Supplementary Figure 5). Hence, diligent pathway mapping revealed amino acid metabolism was identified to be strikingly changed (Figs 3 and 4, Supplementary Table S5).

**Figure 5:**
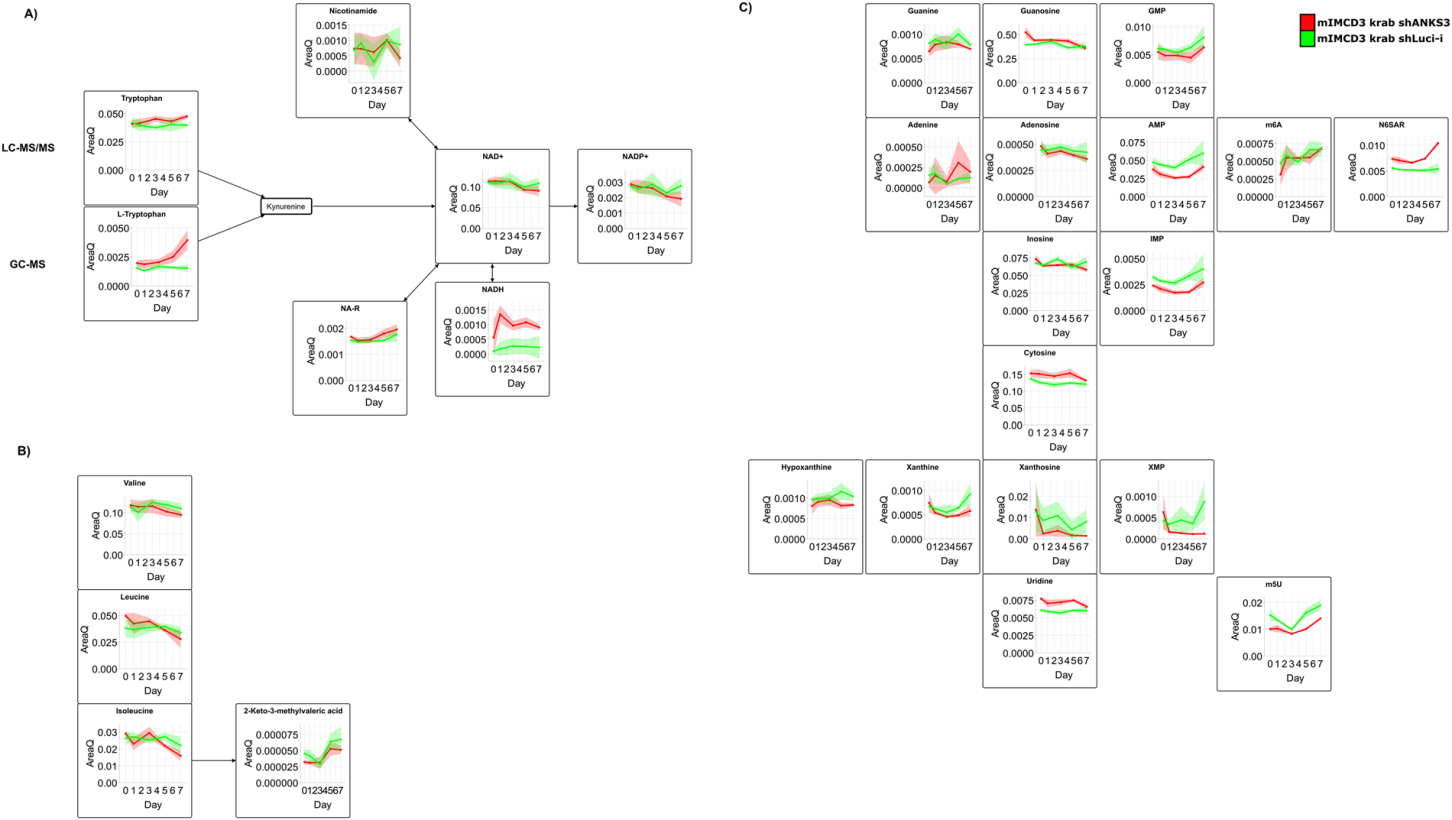
Pathway analysis of selected metabolites. mIMCD3 krab shANKS3 cells were represented in red. Luci-i controls were depicted in green. Shaded areas indicate the standard deviation at each time point. (**A**) Tryptophan-Niacin-NAD pathway analysis. Precursors of NAD^+^ were found to be significantly upregulated whereas reduced levels of NAD^+^ and NADP^+^ were detected. For Trp both GC-MS and LC-MS/MS data was shown. (**B**) Time series analysis of branched-chain amino acids detected by LC-MS/MS. Upon Anks3 depletion all three BCAAs were found at diminished concentrations. 2-keto-3-methylvaleric acid, a degradation product of isoleucine, was found to be upregulated. (**C**) All six unmodified nucleosides were found at lower concentrations after tet treatment. However corresponding bases were not significantly affected. Interestingly, modified nucleosides such as 6-methyladenosine and 5-methyluridine were found to be significantly upregulated.

Many amino acids were differentially regulated upon Anks3 depletion. The essential amino acids threonine (Thr), tryptophan (Trp), lysine (Lys) and phenylalanine (Phe) were shown to be significantly upregulated (Figs 4 and 5A, Supplementary Table S5). In addition to Phe, tyrosine (Tyr) is also upregulated highlighting that Tyr synthesis is catalysed by phenylalanine hydroxylase despite its recycling by proteolysis (Fig. 4, Supplementary Table S5). This might indicate reduced consumption due to diminished protein synthesis. In addition, it has been shown that amino acids are important building blocks for cell mass in proliferating cells^42^. Therefore, accumulation of amino acids can highlight reduced proliferative behaviour of cultured cells.

**Figure 6:**
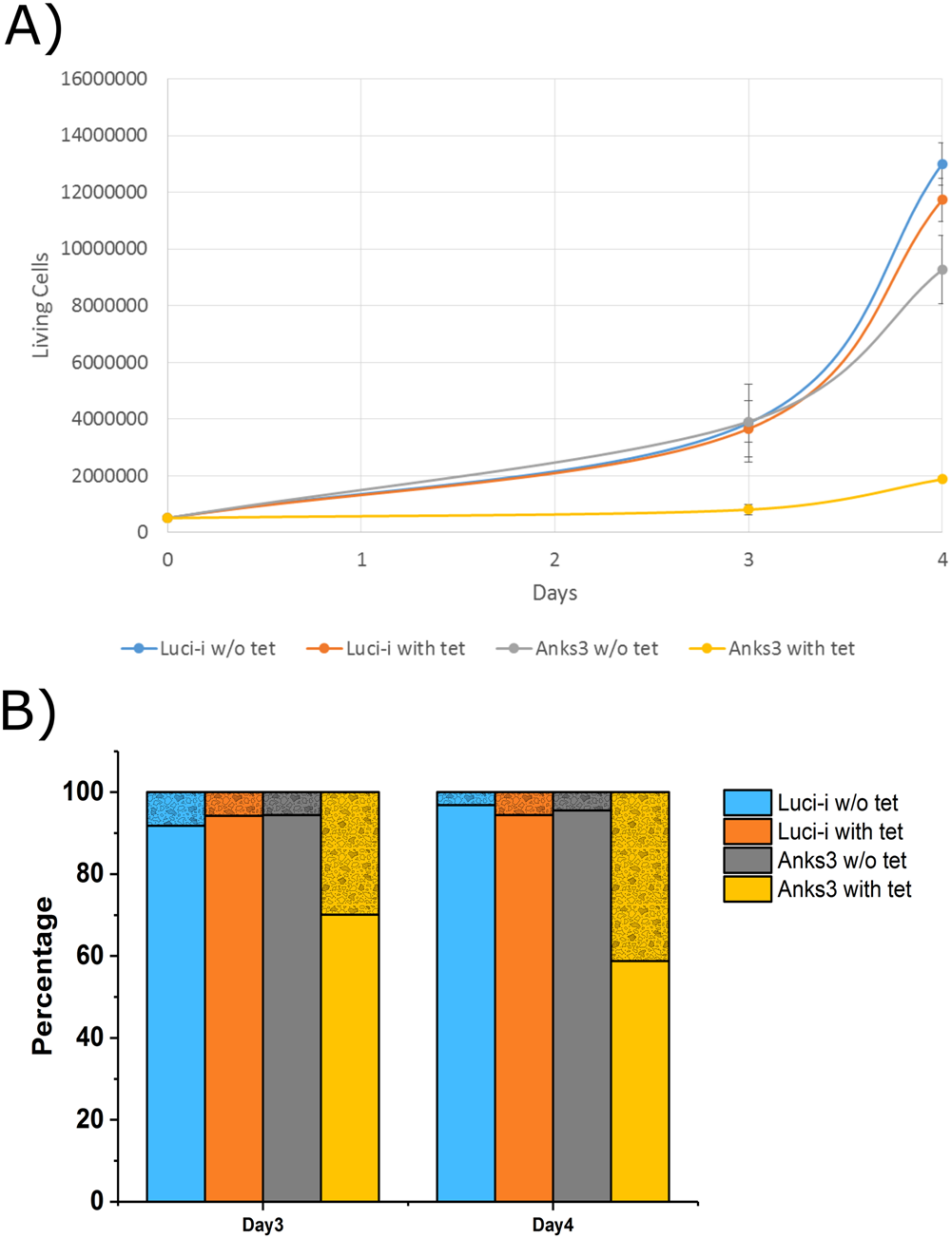
Determination of cell growth and viability. (**A**) Counting assay of living cells. No significant decline of living cells in luciferase control cells upon tet treatment (depicted in orange) compared to untreated cells. Massive decrease of living cells in Anks3 depleted cells (depicted in yellow) compared to untreated cells and treated luciferase control cell line. Error bars indicates standard deviation, n = 3. (**B**) Bar chart of living vs. dead cells stained with trypan blue. A decrease of viability in Anks3 depleted cells (depicted in yellow) was detected compared to control cell lines after Day 3 (70% viability) and Day 4 (60% viability).

Notably, the branched chain amino acids (BCAAs) valine (Val), leucine (Leu) and isoleucine (Ile) were significantly downregulated whereas 2-keto-3-methylvaleric acid, a metabolite of isoleucine, and 3-hydroxyisobutyric acid, an intermediate of valine degradation, were significantly upregulated upon Anks3 protein depletion (Fig. 5B). First, it has been reported that increased BCAAs, especially leucine, play a crucial role in activation of mTORC1 signalling in skeletal muscle cells^43^. Second, it has been reviewed that persistent activation of mTORC1 and S6K by BCAAs might promote insulin resistance and increased levels could be a risk factor of diabetes type 2^44^. Recent findings pointed out that BCAA mediated mTOR hyperactivity enhances cyst development in autosomal dominant polycystic kidney disease resulting in enlarged kidneys^45^. In contrast to autosomal dominant polycystic kidney disease, NPH is characterised by medullary cysts with normal or even slightly reduced kidney size^1,3^. Reduced levels of BCAAs might be causative for reduced mTORC1 activity and subsequently supporting that NPH cyst development is not dependent on mTORC1 mediated cell proliferation. In addition to reduced levels of BCAAs, we elucidated that general proliferation was not affected as shown by approximately 95% ki-67 positive cells (Supplementary Figure 8). However, cell number and viability was strongly affected (Fig. 6). In Anks3 knockdown cells we can highlight a drastically reduced number of living cells (p-value Day 3: 0.0046; p-value Day 4: 0.0010). Also, viability of cells was reduced to 70% after 3 days (p-value 0.06) and 60% after 4 days (p-value 0.001).

Further, intermediates of lipid metabolism such as phosphoethanolamine and cholesterol were differentially regulated upon Anks3 depletion. After depletion of Anks3, upregulation of cholesterol, 1-hexadecylglycerol and 1-octadecylglycerol (monostearylglycerol) were observed whereas phosphoethanolamine was significantly downregulated (Fig. 3, Supplementary Table S5). However, polar extraction with methanol/water is not best suitable for detection of lipids, nonetheless, these alterations might indicate alterations in lipid metabolism. Therefore, application of lipid extraction protocols followed by lipidomics experiments are necessary to highlight whether there are disruptions in lipid metabolism and subsequently membrane composition.

Interestingly, Trp is described to be degraded by aminocarboxymuconate-semialdehyde decarboxylase (ACMSD) via the glutaric acid pathway entering the TCA cycle but also by quinolinate phosphoribosyl-transferase (QPRT) via the niacin pathway for biosynthesis of NAD^+^ and NADP^+^. Both enzymes seem to be highly active in the kidney. However, in rats with adenine-induced renal failure it has been reported, that mainly liver enzymes are responsible for Trp to niacin conversion, although in these rats ACMSD activity in kidney is also reduced, whereas QPRT activity was not affected^46^. In this study, upregulation of Trp was found after induction of Anks3 knockdown, which might point out that Anks3 has an influence on Trp metabolism in mIMCD3 cells (Fig. 5A).

**Figure 7:**
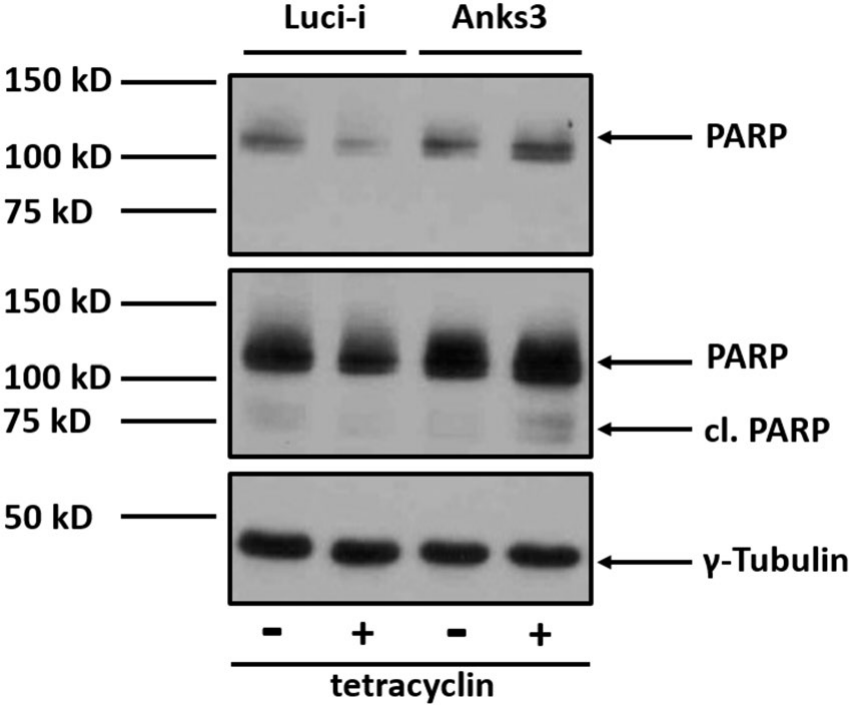
Western blot analysis of PARP1 protein levels. Boxed areas indicate individual western blot exposures. 1 min exposure time was used for upper panel of PARP1. 5 min exposure time was used for lower panel to detect cleaved PARP1. Protein of interest and loading control are stained on the same blot. Increased levels of mature PARP1 protein detectable in tet-treated ANKS3 cells. In addition, higher levels of cleaved PARP1 (cl. PARP) was detectable in Anks3 depleted cells.

Hence, Anks3 depletion also caused decreased levels of NAD^+^ as well as NADP^+^ whereas NADH was not significantly altered (Fig. 5A). Surprisingly, 1-ribosyl-imidazolenicotinamide (NA-R) was found to be slightly upregulated upon Anks3 depletion (p-value: 0.03 and q-value: 0.12) (Fig. 5A). NA-R has been mentioned as precursor of NAD^+^ synthesis by salvage pathway^47,48^. In addition, recent findings highlighted NAD^+^ utilisation as key factor for ARTD-dependent poly(ADP-ribosylation) involved in DNA repair, chromatin remodelling and cell death^47^. The fact that NA-R as salvage precursor tends to be upregulated might support the idea of increased consumption of NAD^+^ upon Anks3 knockdown. Interestingly, glycolysis is not significantly altered as shown by Seahorse data and GC-MS profiling (Fig. 4 and Supplementary Figure 5). Therefore western blot against poly-[ADP-ribose]-polymerase 1 (PARP1) provided higher amounts of both mature PARP1 as well as cleaved PARP1 (Fig. 7). Higher amounts of PARP1 support the idea of increased NAD^+^ consumption due to increased DNA damage^49^. However, accumulation of cleaved PARP1 at 89 kD highlights activation of apoptotic suicide proteases such as caspases^50^. This result also supports the reduced viability stated above (Fig. 6). In addition, tryptophan described as precursor of NAD^+^ synthesis via niacin is upregulated which could either indicate an effect of reduced Trp metabolism or an elevated import of Trp from cell culture media as response to increased NAD^+^ consumption.

It has been reported, that increased DNA damage in eukaryotes subsequently leads to increased production of deoxynucleotides to enable sufficient DNA repair^51,52^. Recently, defective *de novo* synthesis of nucleosides via the non-oxidative pentose phosphate pathway was identified to cause genomic instability and cell death in breast tumours. Additionally, it has been shown, that supplementation of nucleosides increased the intracellular dNTP pool as well as DNA synthesis^53^. In line with that, recent studies underline that decreased levels of nucleotides promote genomic instability and exogenous application of nucleotides reduces DNA damage^54^. Surprisingly, loss of Anks3 yielded a decline of ribonucleosides as determined by LC-MS/MS (Fig. 5C). Furthermore, deoxyinosine (q-value: 0.0007) and deoxyguanosine (p-value: 0.02 and q-value: 0.06) were downregulated (Supplementary Table S5). However, also a trend of reduced deoxyinosine was detected within the Luci-i control cell line (q-value: 0.008). Additionally, increased levels of PARP1 and cleaved PARP1 were detected by western blot indicating higher rates of DNA damage in Anks3 depleted cells (Fig. 7). In line with that, viability was declined after knockdown induction (Fig. 6). In contrast, increased levels of N^6^-Methyladenosine were detected (Fig. 5C). Upon UV-light induced DNA damage, N^6^-methylation of adenosine in RNA was also found to be transiently upregulated and located close to DNA damage sites, however, this effect was never observed using chemicals or y-irradiation^55^. But still, Anks3 knockdown dependent upregulation of N^6^-methyladenosine could also indicate disruption of RNA and DNA metabolism caused by increased DNA damage.

## Conclusions

The present study was aimed to elucidate whether disturbance of NPHP modules by loss of Anks3 protein cause alterations in mitochondrial and cellular metabolism. Up to now, it is the first metabolic characterisation of nephronophthisis phenotype in a mammalian cell culture system. It has been shown, that Anks3 depletion has only mild effects on energy metabolism. However, disruptions of amino acid homeostasis suggests that Anks3 is involved in specific pathways of mitochondrial regulation. Especially, alterations of BCAAs are known to have an influence on important signalling pathway such as mTOR signalling. Interestingly, this study elucidates decreased levels of BCAAs in cells lacking Anks3. Additionally, many other amino acids were significantly upregulated.

Subsequently, it has been observed that Ank3 depletion causes a massive decrease of nucleosides and deoxynucleosides. A decrease of nucleosides can suggest both a higher consumption rate by reason of increased DNA damage or reduced synthesis leading to impaired DNA repair therefore causing increased DNA damage and reduced proliferation. Thus, Anks3 knockdown leads to decreased levels of NAD^+^ and NADP^+^ which may also indicate higher consumption possibly via ARTDs such as PARP1 which is known to be involved in DNA repair and upregulated in Anks3 depleted cells. Hence, Trp and also NA-R tend to be upregulated upon Anks3 depletion supporting the idea of increased NAD^+^ consumption. In combination with reduced cell number, viability as well as elevated PARP1 and cleaved PARP1 levels, the metabolomics data point out increased rates of DNA damage repair and cell death.

Therefore, further proteomic and transcriptomic analyses are important to integrate the findings obtained by this study. Likewise, it is important to monitor whether knockdown of Anks3 interaction partners, such as INVS, results in similar metabolic phenotypes in order to improve the understanding of nephronophthisis on metabolic level.

## Electronic supplementary material

Supplementary Information

dMRM Transitions

IS Matrices

Anova GC-MS Tukey Test

TTest of D0 vs D7
