# Supplementary material for: Metabolic Phenotyping of Anks3 Depletion in mIMCD-3 cells - a Putative Nephronophthisis Candidate
Source: Sci Rep. 2018 Jun 13;8:9022. doi: 10.1038/s41598-018-27389-y (PMC5998149; doi:10.1038/s41598-018-27389-y)
Supplement: Supplementary file 1 — Supplementary Information [file 41598_2018_27389_MOESM1_ESM.pdf]

Metabolic Phenotyping of Anks3 Depletion in mIMCD-3 cells - a Putative Nephronophthisis Candidate

Manuel Schlimpert[1,3,4], Simon Lagies[1,3,4], Vadym Budnyk[2], Barbara Müller[2], Gerd Walz[2], Bernd Kammerer[1,5*]

[1]Center for Biological Systems Analysis, Albert-Ludwigs-University of Freiburg, Germany
[2]Department of Medicine, Renal Division, Albert-Ludwigs-University of Freiburg, Medical Center, Germany
[3]Spemann Graduate School of Biology and Medicine, Albert-Ludwigs-University of Freiburg, Germany
[4]Faculty of Biology, Albert-Ludwigs-University of Freiburg, Germany
[5]University of Freiburg, BIOSS Center for Biological Signaling Studies, Freiburg, Germany

*Corresponding author:

Prof. Dr. Bernd Kammerer, bernd.kammerer@zbsa.uni-freiburg.de, tel. +49 (0)761 203 97137, fax +49 (0)761 203 97177

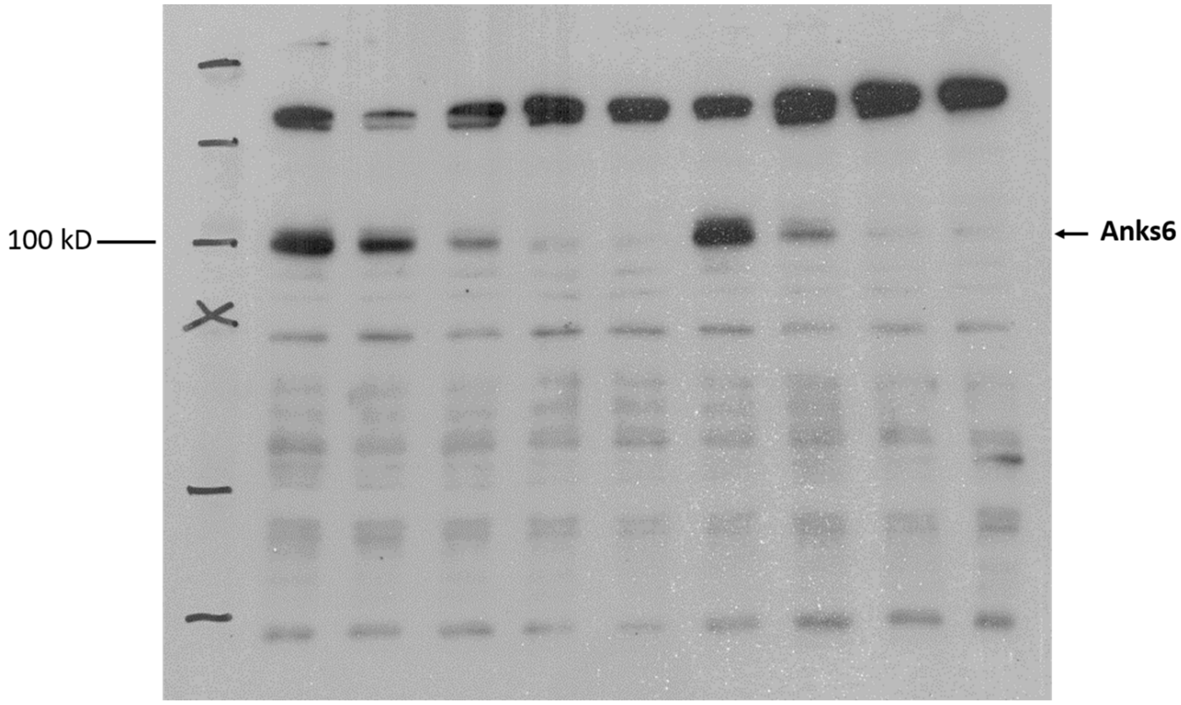

Supplementary Figure 1. Western blot gel of Anks6 staining regarding to Fig. 1B.

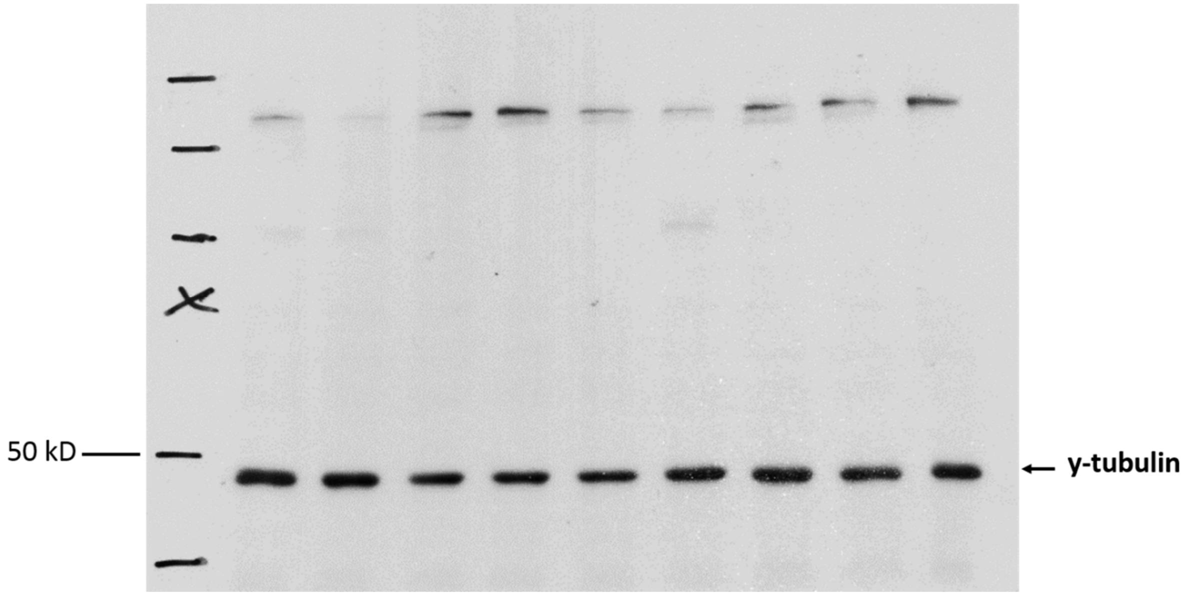

Supplementary Figure 2. Western blot gel of loading control γ-tubulin regarding to Fig. 1A.

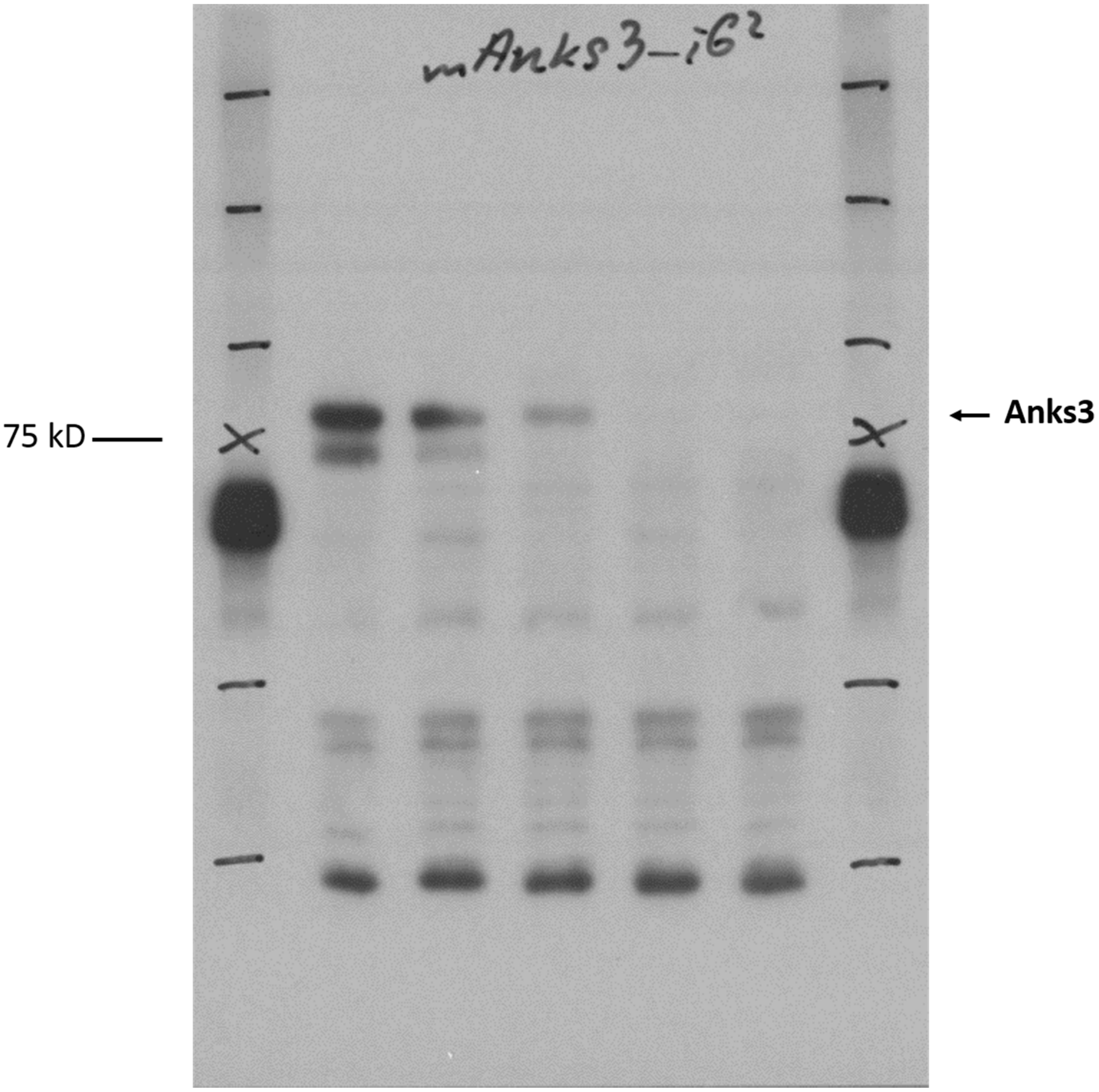

Supplementary Figure 3. Western blot gel of Anks3 staining regarding to Fig. 1B.

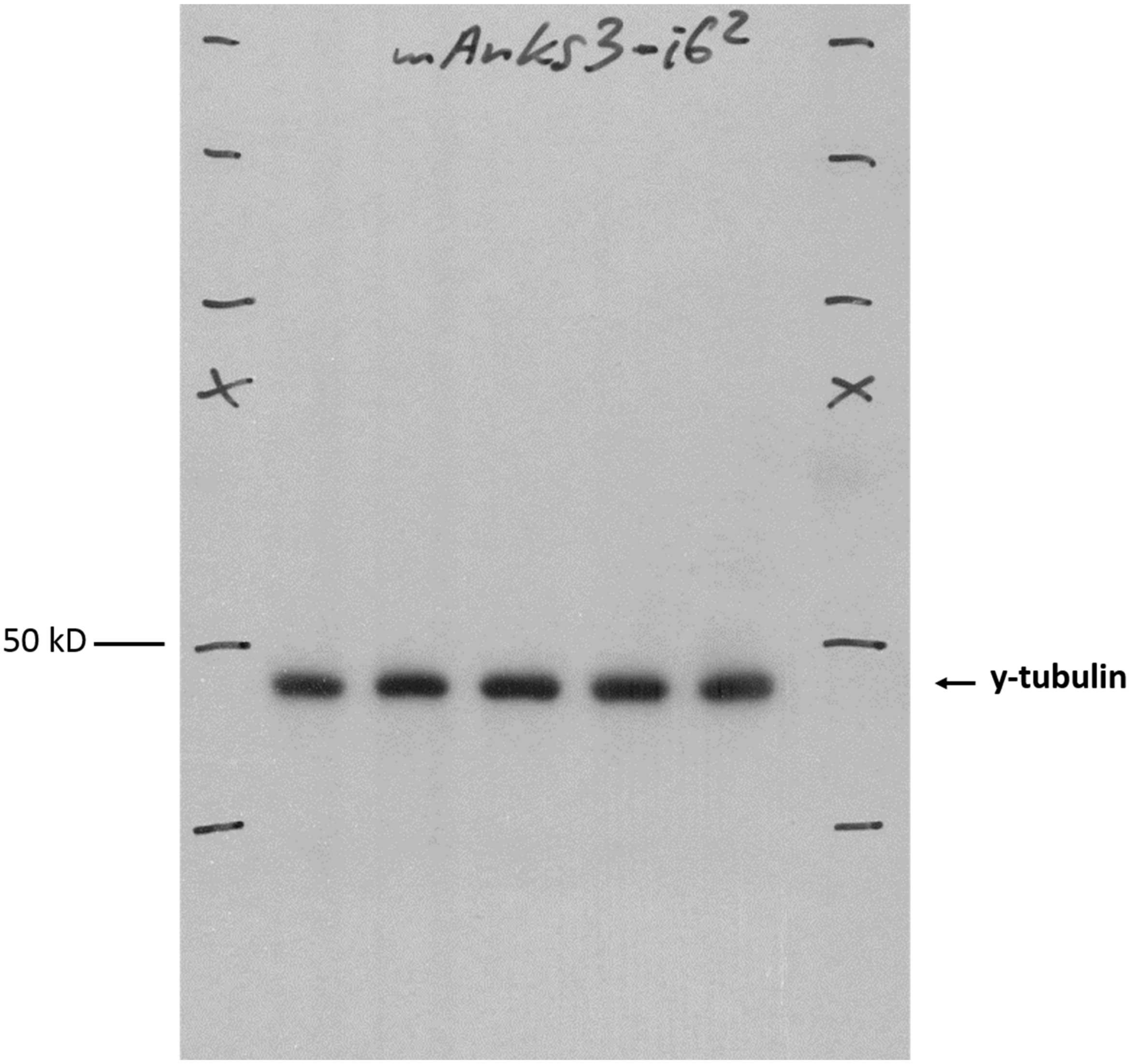

Supplementary Figure 4. Western blot gel of loading control γ-tubulin regarding to Fig. 1B.

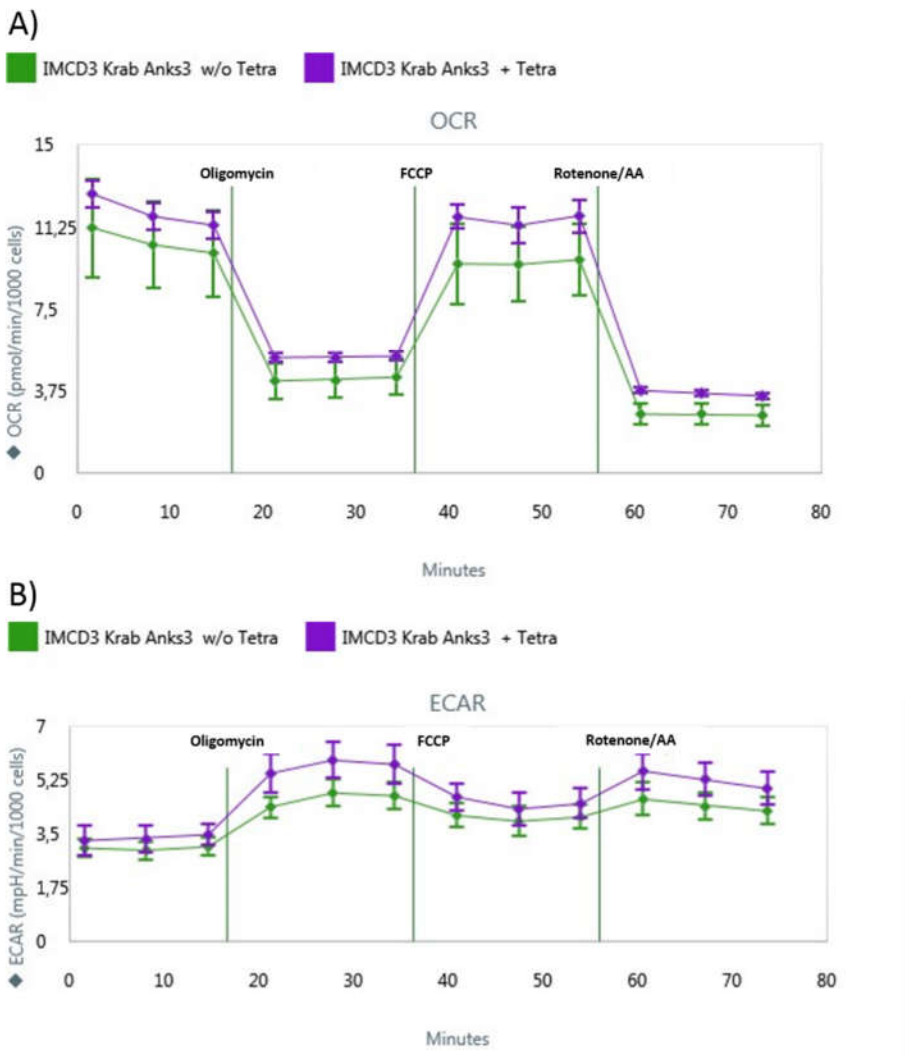

Supplementary Figure 5. Analysis of oxygen consumption and extracellular acidification. A) Oxygen consumption rate (OCR) and B) extracellular acidification rate (ECAR) of IMCD3 krab shAnks3 were measured in triplicates. Knockdown of Anks3 has no significant effect on OCR and ECAR highlighting no significant impact on glycolytic flux in Anks3 depleted cells.

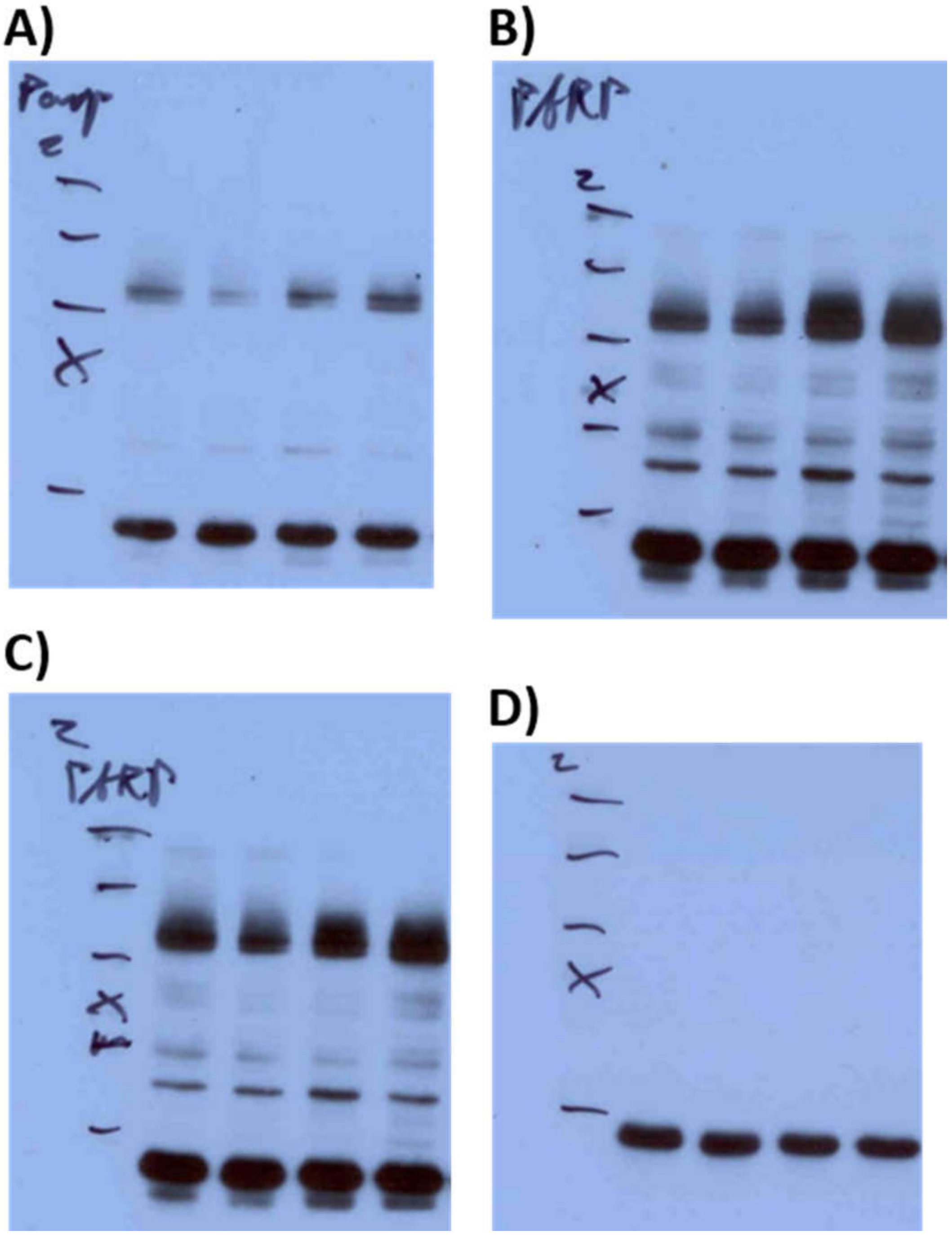

Supplementary Figure 6. Original western blot membranes. Marker band at 75 kD is represented by X. A) PARP antibody exposure time 1 min provides nice staining for uncleaved PARP. B) PARP antibody exposure time 5 min provides nice staining for cleaved PARP at 89 kD, overexposure of uncleaved PARP. C) PARP antibody exposure time 3 min provides detectable staining for uncleaved PARP at 89 kD in ANKS3 KD cells (lane 4), overexposure of uncleaved PARP. D) γ-tubulin antibody exposure 2 s.

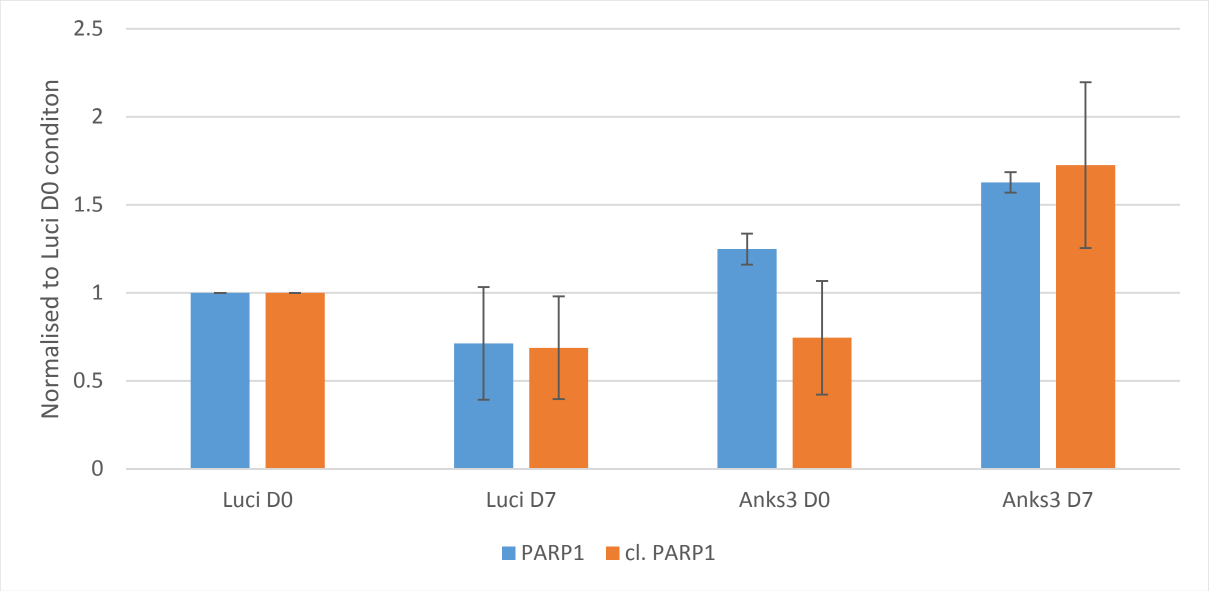

Supplementary Figure 7. Quantification of Western Blots. Western blots against PARP1 and cleaved PARP1 were performed in duplicates. Intensities were normalised to tubulin as loading control and subsequently to untreated luciferase control cell line. Error bars indicate standard deviation.

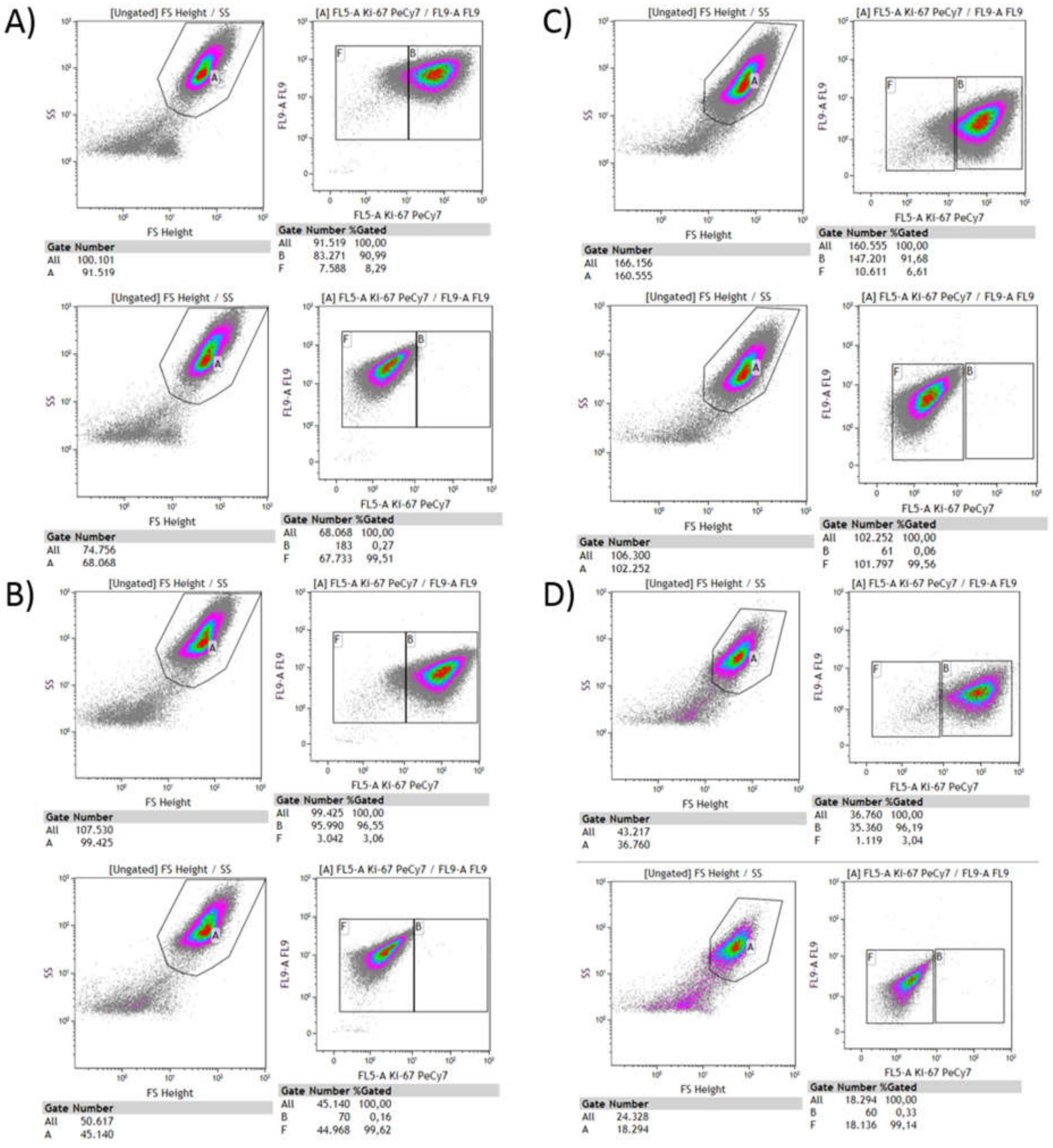

Supplementary Figure 8. KI-67 staining of mIMCD3 cells. Gating for living cells is depicted on the left panels. Right panels highlight negative isotope controls (bottom) and ki-67 positive cells (top). A) ki-67 staining for mIMCD3 krab shluci-i without tet. B) ki-67 staining for mIMCD3 krab shluci-i with tet. A) ki-67 staining for mIMCD3 krab shAnks3 without tet. B) ki-67 staining for mIMCD3 shkrab Anks3 with tet. In all conditions over 90% ki-67 positive cells were observed. However, in Anks3 depleted cells (D) much lower amount of living cells could be gated. Higher amounts of dead cells were observed in FS/SS blot.

Supplementary Table 1. Schematic overview of experimental cell culture setup used in this study. MC stands for medium change. 0.125 µg/mL indicates treatment with tetracycline.

Cell Lines:
mIMCD3 shRNA Anks3
mIMCD3 shRNA Luci-i

Seeded Cells

**Day -1**	**Day 0**	**Day 2**	**Day 4**	**Day 6**	**Day 7**
**0.5 x 10^6**	**0.125 µg/mL**	MC	MC		**Harvest**
	MC	**0.125 µg/mL**	MC		**Harvest**
	MC	MC	**0.125 µg/mL**		**Harvest**
	MC	MC	MC	**0.125 µg/mL**	**Harvest**
**0.5 x 10^6**	MC	MC	MC		**Harvest**
